# Supplementary material for: Direct observation of mode-specific phonon-band gap coupling in methylammonium lead halide perovskites
Source: Nat Commun. 2017 Sep 25;8:687. doi: 10.1038/s41467-017-00807-x (PMC5612932; doi:10.1038/s41467-017-00807-x)
Supplement: Supplementary file 1 — Supplementary Information [file 41467_2017_807_MOESM1_ESM.pdf]

Supplementary Figures

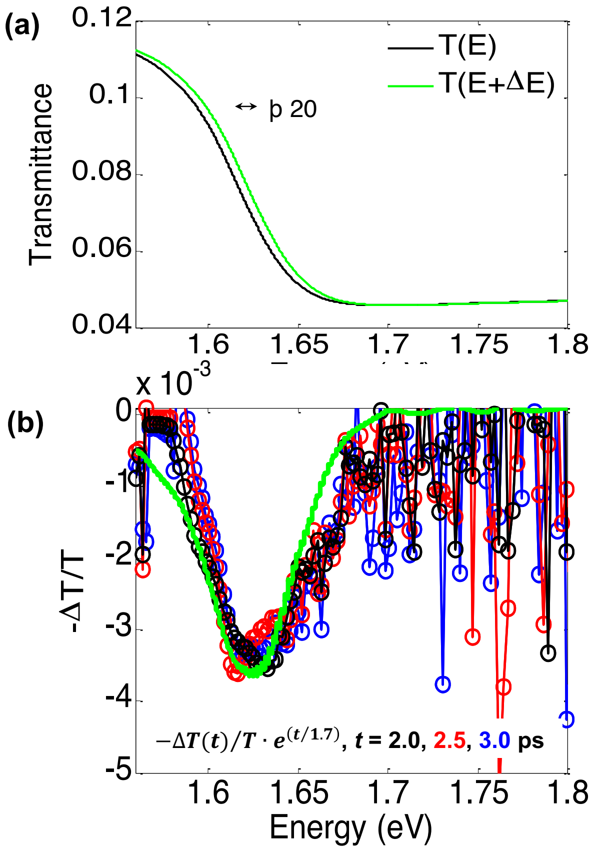

Supplementary Figure 1. (a) Linear optical transmission spectrum of the sample (black) and a blue-shifted transmission spectrum (green). The extent of the optical band gap shift (ΔE) was 20-fold magnified for visual clarity (green). (b) Difference between the measured and shifted transmission spectrum of (a, solid green line) together with (THz excitation induced) transient spectra at long delay times (at $t > 1$ ps, Fig. 1b) extrapolated to $t = 0$.

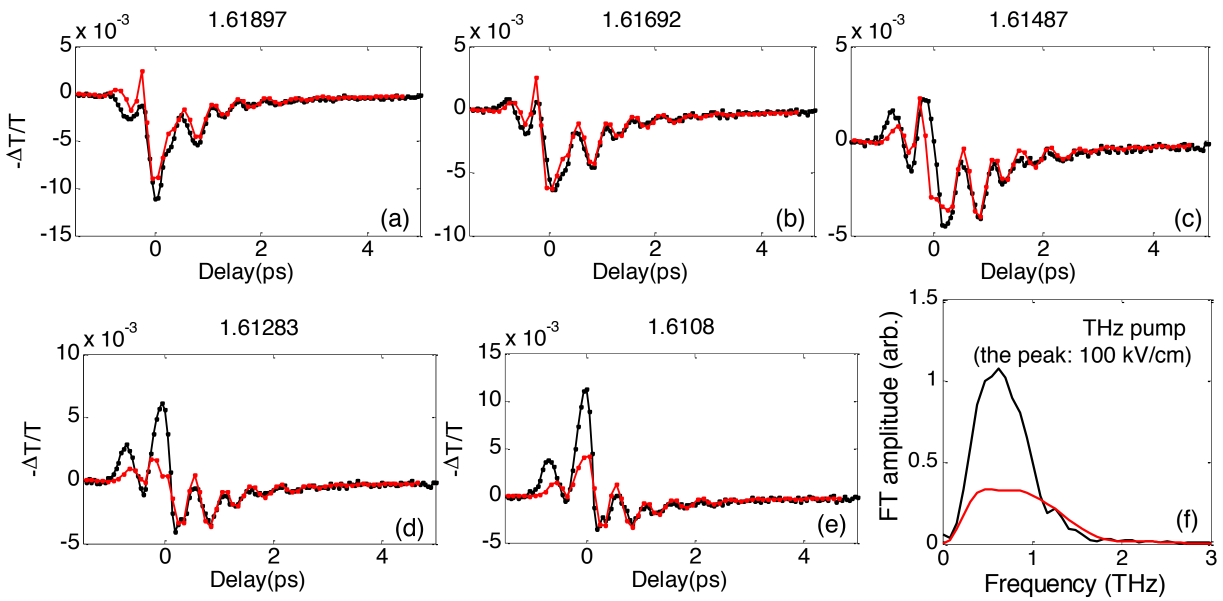

Supplementary Figure 2. THz pump-optical probe experiments using different spectral contents of the THz pump pulses with the same peak field strength. (a-e) Time traces of the differential spectra $(-\Delta T/T)$ at different probe energies (eV) for a narrow (black traces) and a broader (red traces) THz excitation bandwidth (f) The Fourier transform of two different THz pump pulses. The pump probe signals at $t > 1$ ps for different THz bandwidths coincide (both regarding shape and amplitude).

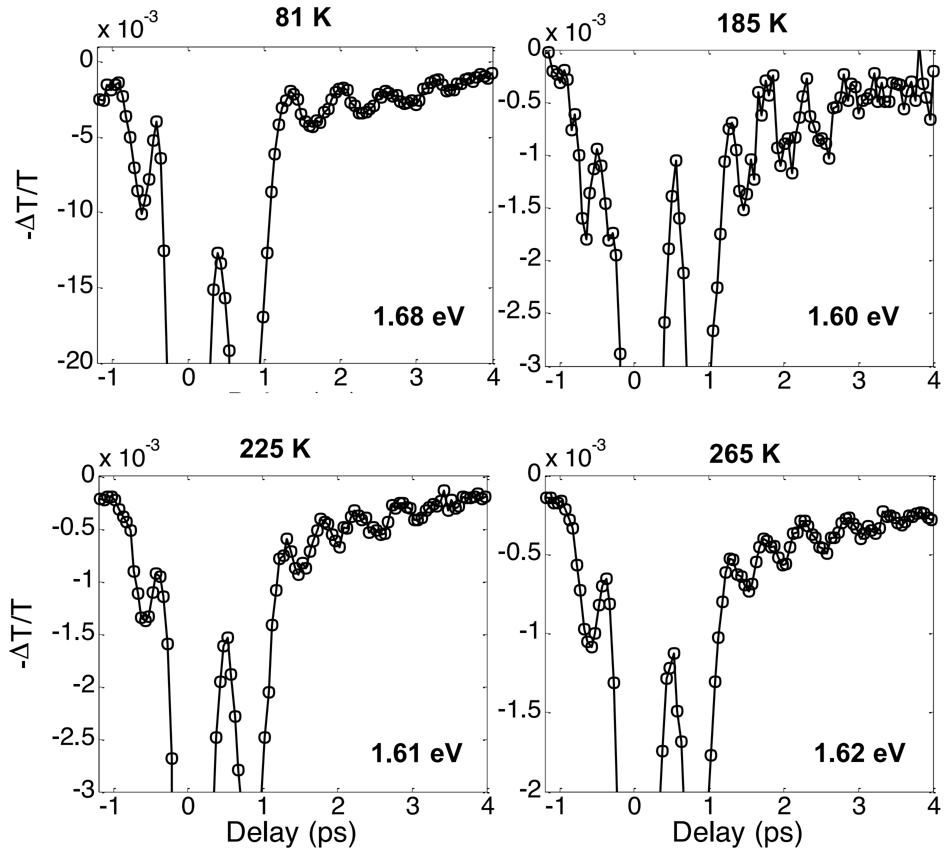

Supplementary Figure 3. Time traces of THz pump visible probe signals at near gap probe energies as a function of sample temperature (1.68 eV for 81 K, 1.60 eV for 185 K, 1.61 eV for 225 K, and 1.62 eV for 265 K)

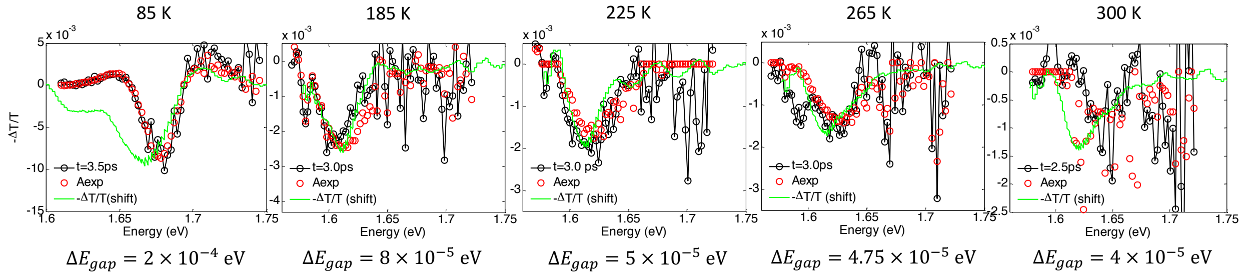

Supplementary Figure 4. Comparisons of the blue shift of the transmission curve (green), the differential spectra at t = 3.0 ps (3.5 ps at 85 K, black) multiplied by a factor of $1/e^{(t/\tau_{11})}$ (See Supplementary Note 1), and the amplitude spectra of the exponentially decaying component obtained from fitting a damped oscillator to the transient signals.

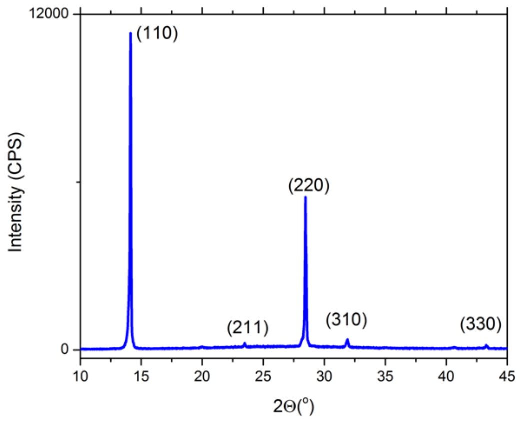

Supplementary Figure 5. XRD pattern of the $MAPbI_3$ perovskite film

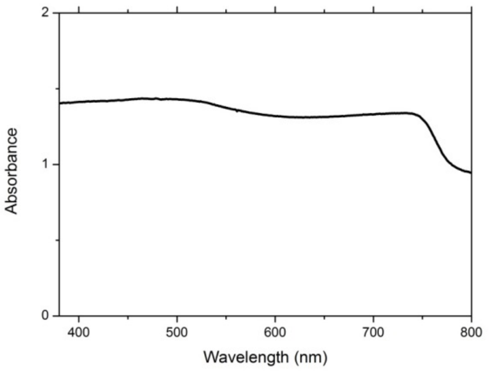

Supplementary Figure 6. Absorbance spectrum of the freshly prepared $MAPbI_3$ perovskite film

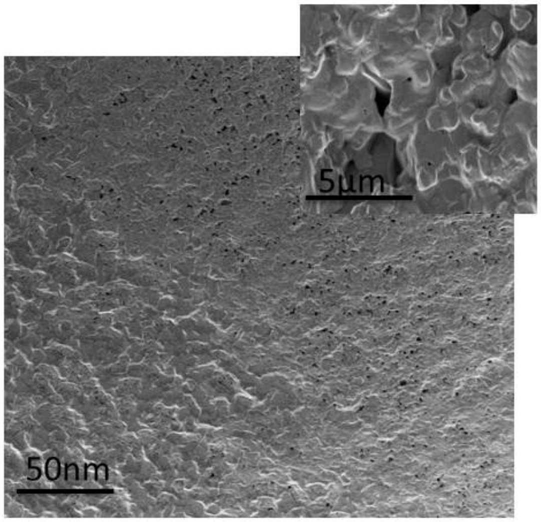

Supplementary Figure 7. SEM images of the $MAPbI_3$ perovskite film

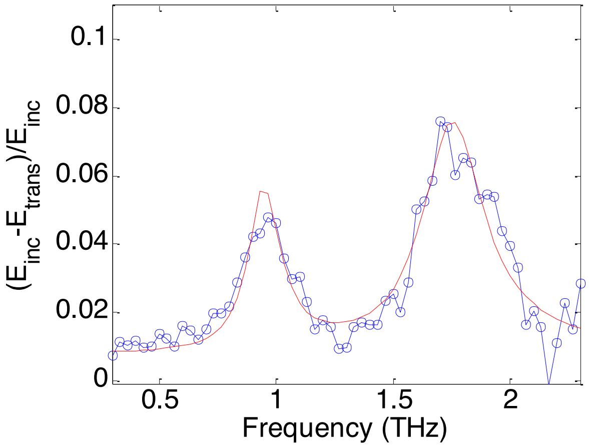

Supplementary Figure 8. THz absorption spectrum of the $MAPbI_3$ perovskite film (blue circles). The solid red line shows a fit using two Lorentzian bands to the spectrum.

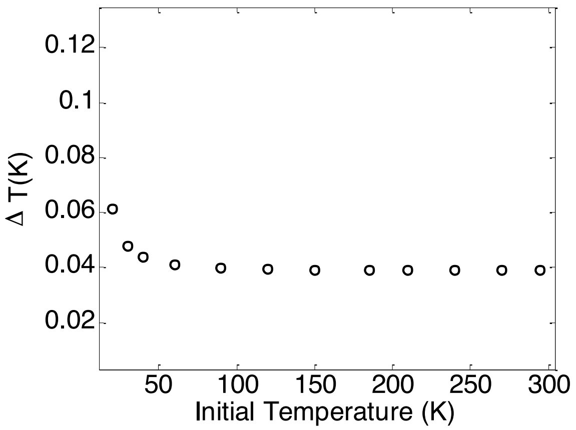

Supplementary Figure 9. The estimated phonon temperature rise (ΔT_{ph}) at different initial temperatures with the values of $N_{\mathrm{tot}} = 2.7894 \cdot 10^{15}, \Delta E_{\mathrm{Abs}} = 1.4923 \cdot 10^{-9}$ $[J], \nu = 0.94\times10^{12}$. In this high temperature range (185~300 K), ΔT_{ph} is not sensitive to the initial temperature.

Supplementary Table 1

Temperature	$1/\Gamma_{11}$ (ps)	$1/\Gamma_{20}$ (ps)	ω_{20} (THz)
295 K	1.71±0.16	0.78±0.14	1.95±0.05
265 K	2.0±0.7	1.2±0.4	1.88±0.08
225 K	1.9±0.7	1.0±0.4	1.83±0.08
185 K	1.7±0.7	1.0±0.4	1.80±0.07
81 K	2.1±0.9	1.4±0.4	1.55±0.04

Supplementary Table 1. Fit parameters obtained by fitting a damped oscillator model (eq. 5 of the main manuscript) to the pump probe signals at t >1.3 ps

Supplementary Note 1

The basic parameters from our experimental condition are as follows:

The THz beam has a diameter of ~ 1 mm at the focal point (sample position). Together with the sample thickness of L: ~ 300 nm this amounts to a volume of $\pi \cdot (0.5 \cdot 10^{-3})^2 \cdot 300 \cdot 10^{-9}\ \ (m^3)$ that is exposed to the THz pulse.

The incident THz pulse energy per unit area (m^2), with peak field amplitude of 1.0×10^7 V/m is given by

$$\int_{-\infty}^{\infty} \epsilon_0 cn|E_{\mathrm{THz}}(t)|^2 dt = 0.0993\ [J/m^2] = 1/2\pi \int_{-\infty}^{\infty} \epsilon_0 cn|E_{\mathrm{THz}}(\omega)|^2 d\omega$$

(Supplementary Equation 1)

, where ω is the angular frequency, ϵ_0 the vacuum permittivity, c the speed of light, and n the refractive index of air (=1.0).

The THz pulse energy absorbed by the sample per unit area (m^2) amounts thus to:

$$1/2\pi \int_{-\infty}^{\infty} \epsilon_0 cn\left(E_{THz}^2(\omega) - E_{trans}^2(\omega)\right) d\omega = 1.9 \cdot 10^{-3} [J/m^2]$$

(Supplementary Equation 2)

, where the absorption cross-section of the 1 THz mode in this sample was determined from the THz absorption spectrum (Supplementary Figure 8).

At long delay times, the absorption of THz pulses will lead to a transient rise of the average temperature (ΔT_{avg}) in the focal volume. We estimated ΔT_{avg} by using reported values of the heat capacity of $MAPbI_3$ perovskites (at 295 K: ~190 J K^{-1} mol^{-1}).[1] The molar density was determined from the lattice constants of the tetragonal phase (the space group of *I4/mcm* with lattice constants of a = b = 8.98 Å, c = 12.57 Å for a 48-atom unit cell[2]) to

$$\rho = \frac{4}{N_A \cdot (8.98\times 8.98\times 12.57\times 10^{-30})}\ \ [mol\ m^{-3}]$$

(Supplementary Equation 3)

Accordingly, absorption of the THz pulse will result in an average temperature rise (assuming equilibrium distribution of the THz energy over all available modes) of:

$$\Delta T_{\mathrm{avg}} = \frac{\text{Absorbed Pulse Energy per unit area} \times \text{Focal size}}{\text{Heat capacity} \times \text{Molar density} \times \text{Optical volume}}$$

$$= \frac{1.9 \cdot 10^{-3}\ [J\ m^{-2}] \times \pi \cdot (0.5 \cdot 10^{-3})^2\ [m^2]}{190\ [J\ K^{-1}\ mol^{-1}] \times \frac{4}{N_A \cdot (8.98\times 8.98\times 12.57\times 10^{-30})}\ [mol\ m^{-3}] \times \pi \cdot (0.5 \cdot 10^{-3})^2 \cdot 300 \cdot 10^{-9} [m^3]}$$

$$\sim 5.1\times 10^{-3}\ \mathrm{K}$$

(Supplementary Equation 4)

Assuming the 1 THz phonon (Pb-I-Pb bending) mode to be a localized oscillator, the number of Pb-I-Pb bonds in a unit cell is 3 (number of edges (12) divided by number of sharing unit cells (4)). We note that the number of total degrees of freedom in one unit cell, which is three times the number of atoms (12), is 36. Thus, the total number of oscillators in the optical volume,

$$N_{\text{tot}} = \text{Oscillator number density} \times \text{Optical volume}$$

$$= \frac{3\times4}{8.98\times8.98\times12.57\times10^{-30}} \cdot \pi \cdot (0.5 \cdot 10^{-3})^2 \cdot 300 \cdot 10^{-9} = 2.7894 \cdot 10^{15}$$

(Supplementary Equation 5)

and the THz energy that is absorbed by the phonon,

$$\Delta E_{\text{Abs}} = \text{Absorbed Pulse Energy per unit area} \times \text{Focal size}$$

$$= 1.9 \cdot 10^{-3}\ [J\ m^{-2}] \times \pi \cdot (0.5 \cdot 10^{-3})^2\ [m^2] = 1.4923 \cdot 10^{-9}\ [J]$$

(Supplementary Equation 6)

Supplementary References

1. Knop, O., Wasylishen, R. E., White, M. A., Cameron, T. S. & Oort, M. J. M. Van. Alkylammonium lead halides. Part 2. CH 3 NH 3 PbX 3 (X = Cl, Br, I) perovskites: cuboctahedral halide cages with isotropic cation reorientation. *Can. J. Chem.* **68,** 412–422 (1990).
2. Ong, K. P., Goh, T. W., Xu, Q. & Huan, A. Structural Evolution in Methylammonium Lead Iodide CH3NH3PbI3.(1) Ong, K. P.; Goh, T. W.; Xu, Q.; Huan, A. J. Phys. Chem. A 2015, 119 (44), 11033. *J. Phys. Chem. A* **119,** 11033–8 (2015).
